# Performing point-of-care molecular testing for SARS-CoV-2 with RNA extraction and isothermal amplification

**DOI:** 10.1371/journal.pone.0243712

**Published:** 2021-01-11

**Authors:** Pierre Garneret, Etienne Coz, Elian Martin, Jean-Claude Manuguerra, Elodie Brient-Litzler, Vincent Enouf, Daniel Felipe González Obando, Jean-Christophe Olivo-Marin, Fabrice Monti, Sylvie van der Werf, Jessica Vanhomwegen, Patrick Tabeling

**Affiliations:** 1 ESPCI PSL, CBI, IPGG, Paris, France; 2 Institut Pasteur, Paris Cedex, France; University of Helsinki, FINLAND

## Abstract

To respond to the urgent need for COVID-19 testing, countries perform nucleic acid amplification tests (NAAT) for the detection of SARS-CoV-2 in centralized laboratories. Real-time RT—PCR (Reverse transcription—Polymerase Chain Reaction), used to amplify and detect the viral RNA., is considered, as the current gold standard for diagnostics. It is an efficient process, but the complex engineering required for automated RNA extraction and temperature cycling makes it incompatible for use in point of care settings [1]. In the present work, by harnessing progress made in the past two decades in isothermal amplification and paper microfluidics, we created a portable test, in which SARS-CoV-2 RNA is extracted, amplified isothermally by RT—LAMP (Loop-mediated Isothermal Amplification), and detected using intercalating dyes or fluorescent probes. Depending on the viral load in the tested samples, the detection takes between twenty minutes and one hour. Using a set of 16 pools of naso-pharyngal swab eluates, we estimated a limit of detection comparable to real-time RT-PCR (i.e. 1 genome copies per microliter of clinical sample) and no cross‐reaction with eight major respiratory viruses currently circulating in Europe. We designed and fabricated an easy-to-use portable device called “COVIDISC” to carry out the test at the point of care. The low cost of the materials along with the absence of complex equipment will expedite the widespread dissemination of this device. What is proposed here is a new efficient tool to help managing the pandemics.

## Introduction

Nucleic Acid Amplification Tests (NAATs) detect the presence of pathogen genomes in infected samples through specific amplification of theirnucleic acids,. They are characterized by a low limit of detection (LoD),allowing detection of viral loads as low as 1–100 genome copies per microliter of sample, and an excellent analytical specificity. There are different types of NAATs for severe acute respiratory syndrome coronavirus 2 (SARS-CoV-2) testing but today, RT-PCR (Reverse Transcription Polymerase Chain Reaction), coupled to extraction, is considered as the gold standard for diagnostic laboratories.

For getting high-throuput, RT-PCR, due to an inherent complexity has to be performed in parallelized tests in centralized laboratories. This generates logistics issues, long delivery times, (several days in practice) and excessive costs for developing countries. Over the last ten years, with the advent of isothermal amplification technologies [2], hope has been raised to perform NAATs at the point of care, with portable devices much cheaper than extraction/RT-PCR platforms, and with comparable performances.

In this context, NAATs coupling isothermal amplification to paper microfluidics have been developed in laboratories [3–16]. These systems possess a potential, but owing a number of limitations (moderate practicability, absence of performing sample preparation in most cases, incomplete evaluation of the clinical and analytical performances) these devices have not reached a state, yet, where they can be commercialized. Thereby, as SARS-CoV-2 started to propagate, the technology was not ready to face the request of large scale global testing. Recently, commercial isothermal detection assays have been proposed [17]. However, because of substantial cost, limited throughput and moderate performances, they have not taken off. Isothermal detection of SARS-CoV-2has been reported in the literature, but these assays do not represent yet an alternative to PCR testing, often because they do not include the extraction of the nucleic acids (NA), which affects their sensitivity [18–21].

The molecular tests we report here are portable and low cost. The new feature is that they combine NA extraction, RT-LAMP and naked eye visualization capability on the same device. Teir performances are comparable to the extraction/RT-PCR method. The practical device we created (COVIDISC) is adapted for the detection of SARS-CoV-2 at the point of care.

## Materials and methods

### Device fabrication

**Fig 1:**
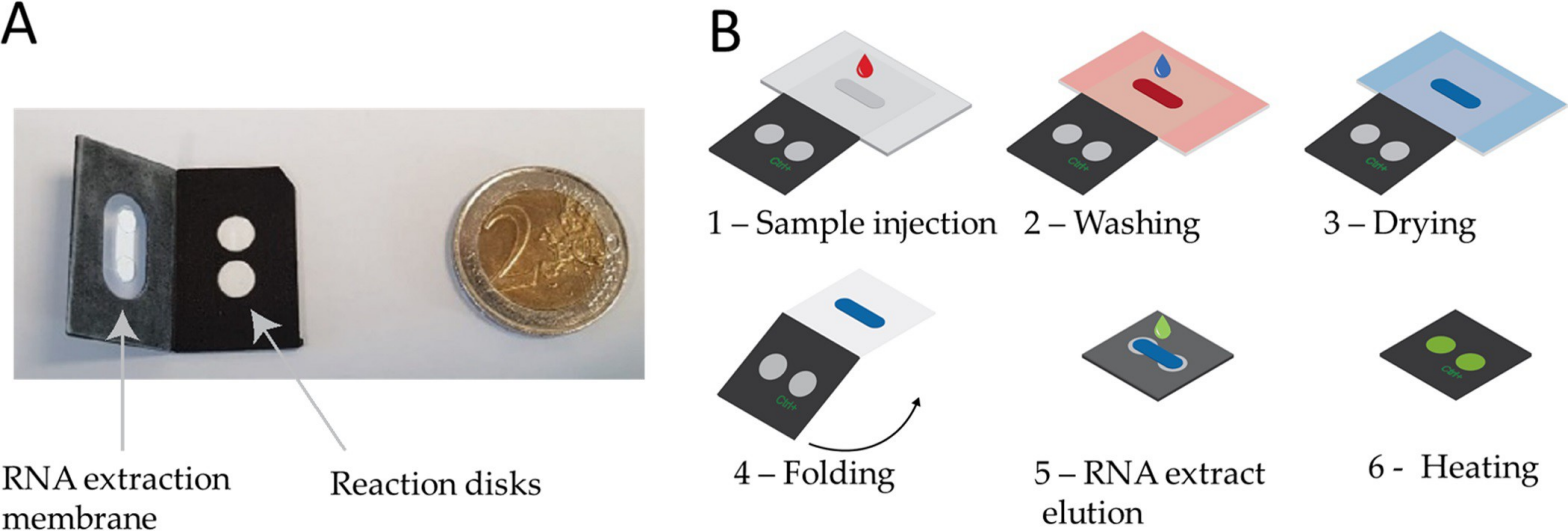
Laboratory device and workflow. A: Two sheets of polypropylene (black) in which pretreated pieces of glass fiber (in white) are incorporated. B: Mode of operation. 1 –The sample, in which the virus has been lysed, is injected onto the extraction membrane. 2 –Washing. 3 –Drying of the extraction membrane. 4 –Folding of the device in such a way that the extraction membrane comes into contact with the two reaction disks. On both disk is freeze-dried the LAMP mix and primers (Covid-19 and human 18S RNA for the test and control disk respectively) permiting reverse transcription and amplification. 5 –Elution of the RNA from the capture membrane to the reaction disks, then, sealing with PCR tape 6 –Heating at 65°C. Read-out in real time with an intercalating agent (SYTO82).

The device is shown on Fig 1A. Two sheets of black polypropylene (Polydis, 800μm thick), covered on one side by PCR tape (thermal Seal RT2R, Sigma Aldrich) two centimeters in size, incorporate pretreated laser-cut membranes: one (oblong form: 2mm x 10mm) for nucleic acid extraction, and the two others (circular form: 5.4 mm diameter) for the RT-LAMP reactions. Capture membrane and reaction membranes are binder-free glass fiber provided by Cytiva. COVIDISC devices are 3D-printed using a Poly lactic acid (PLA) resin, printed at 115°C at 90mm/second speed, 0.15 mm layer thickness. The diameter of the COVIDISC is 65 mm.

### RT-LAMP reaction

RT-LAMP primer sequences have been retrieved from published work [22–25]. The freeze dried reaction mix is composed of 8U GspSSD 2.0 polymerase (OptiGene), 0.5 U AMV-RT (Life Sciences Advanced Technologies), 4 mM MgSO4, 0.4 mM of each dNTPs (dATP, dTTP, dGTP, dCTP), 5μM SYTO-82 intercalating dye (Invitrogen), 0.2 μM of F3/B3 primers, 0.8 μM FIP/BIP primers, 0.4 μM of LF/LB primers, 1M Betain and 10% trehalose. Volumes of 18μL reaction mix are pipetted onto reaction membranes and freeze as previously described in [24].

### Sample preparation

AVL buffer was used for sample lysis (QIAGEN). Rinsing buffer was made according to previously described recipe [23]. Elution was performed using isothermal reaction buffer (iBuffer) provided by OptiGene. For sample and washing steps, a cellulose absorbant pad (Whatman CF7) was used for downstream capillary pumping.

### Biological samples

Naso-pharyngal swabs collected from suspected COVID-19 cases and eluted in Universal Viral Transport medium (UTM) were provided by Institut Pasteur biobank. To prepare a reference set of SARS-CoV-2 positive samples, swab eluates presenting similar SARS-CoV-2 viral loads (genome quantities) were mixed together to obtain eight pools with concentrations spanning the range of viral loads detected in COVID-19 patient samples, as measured with the real-time duplex qRT-PCR (targeting the RdRP gene) developed by the French National Reference Centre [26,27]. In terms of viral loads, the pools span a range of RT-PCR cycle threshold (Ct) values extending from 15 to 38, i.e from 10^8^ to 1 copies per microliter of clinical sample (See S3 Fig in S1 File for the conversion between Ct values and viral load.). Each pool was tested in duplicate to determine the limit of detection of the method. To determine the reaction specificity, we used eight additional pools of clinical naso-pharyngal samples, not infected by SARS-CoV-2, but collected from patients infected by other respiratory pathogens: respiratory syncytial virus (RSV) A and B, influenza A (H1) virus, influenza A (H3) virus, influenza B Victoria (BVIC), Yamagata lineages (BYAM), human rhinovirus (HRV) and human metapneumovirus (hMPV). Swab eluates presenting high viral loads (above 1000 copies/μL) were used to prepare these pools. In addition, 5 SARS-CoV-2 negative naso-pharyngal eluates were used as control.

### Operating method

Fig 1B shows the workflow. The sample is mixed with a lysis buffer. The role of this buffer is both to lyze the virus and enable the nucleic acid extraction process. The principle of the nucleic acid extraction was described by Boom [28]. Essentially, the sample is mixed with a salt and ethanol buffer. Concentrated salt inverts the ζ-potential of the matrix (from negative to positive). With that inversion, the negatively charged nucleic acids (RNA and DNA) are captured. The other sample components (proteins, membranes, etc.) are precipitated by ethanol and transported downstream, through the extraction disk. In practice, 100 μL of the sample, are mixed with 200μL AVL buffer (Qiagen), and incubated for 10 minutes. A volume of 200μL pure ethanol is then added and the solution is introduced in the extraction membrane and washed with a washing buffer according to following reference [23,29,30]. In order to manage the buffers flowing through the capture membrane, a wick is placed in contact with it to pump the fluids by capillarity. The absorbent is further thrown away. After drying for 15 minutes, the device is folded so as to place the extraction membrane in contact with the two reaction membranes. RNA is eluted by using Optigene standard isothermal buffer (iBuffer). In this process, the buffer releases the captured RNA from the extraction membrane, and, owing to the action of capillarity, penetrates in the two reaction disks. On both disks, the freeze-dried reaction mixis hydrated, and mixes with the RNA extracts. Upon temperature rise to 65°C (by maintaining the paper device on a hot plate), reverse transcription and amplification are performed. We use real-time RT-LAMP. Its kinetics is measured by tracking the fluorescence emission of a DNA intercalating dye (SYTO-82). The target is Orf1ab (primers are displayed in S1 Table in S1 File [19]) and ARN 18S as internal sample processing control and sample quality control. Measuring the kinetics of the amplification allows to estimate the noise level and control the shapes of the amplification curves. The noise level should be low (a deficient extraction often generates noise on such curves) and the amplification curves should be sigmoidal. Any deviation from these expectations may signal the presence of an artifact. Indeed, for on-field applications, kinetic studies are not necessary. Only end-point data (i.e after one hour amplification) is needed for providing a YES/NO answer.

### Ethical documentation

Samples used in this study were collected as part of approved ongoing surveillance conducted by the National Reference Center for Respiratory Viruses (NRC) at Institut Pasteur (WHO reference laboratory providing confirmatory testing for COVID-19). The lab investigations described in this article were carried out in accordance with the General Data Protection Regulation (Regulation (EU) 2016/679 and Directive 95/46/EC) and the French data protection law (Law 78–17 on 06/01/1978 and Décret 2019–536 on 29/05/2019) which does not require a review by an ethics committee for the secondary use of samples collected for healthcare purposes. In such case, the secondary use for research is authorized if the persons have been informed of such secondary use (article L.1211-2 of the French Public Health Code). A specific request for an information derogation has been submitted to a competent national ethics committee for the tested control samples for which provision of such information for the secondary use of the samples could not be ascertained.

## Results

**Fig 2:**
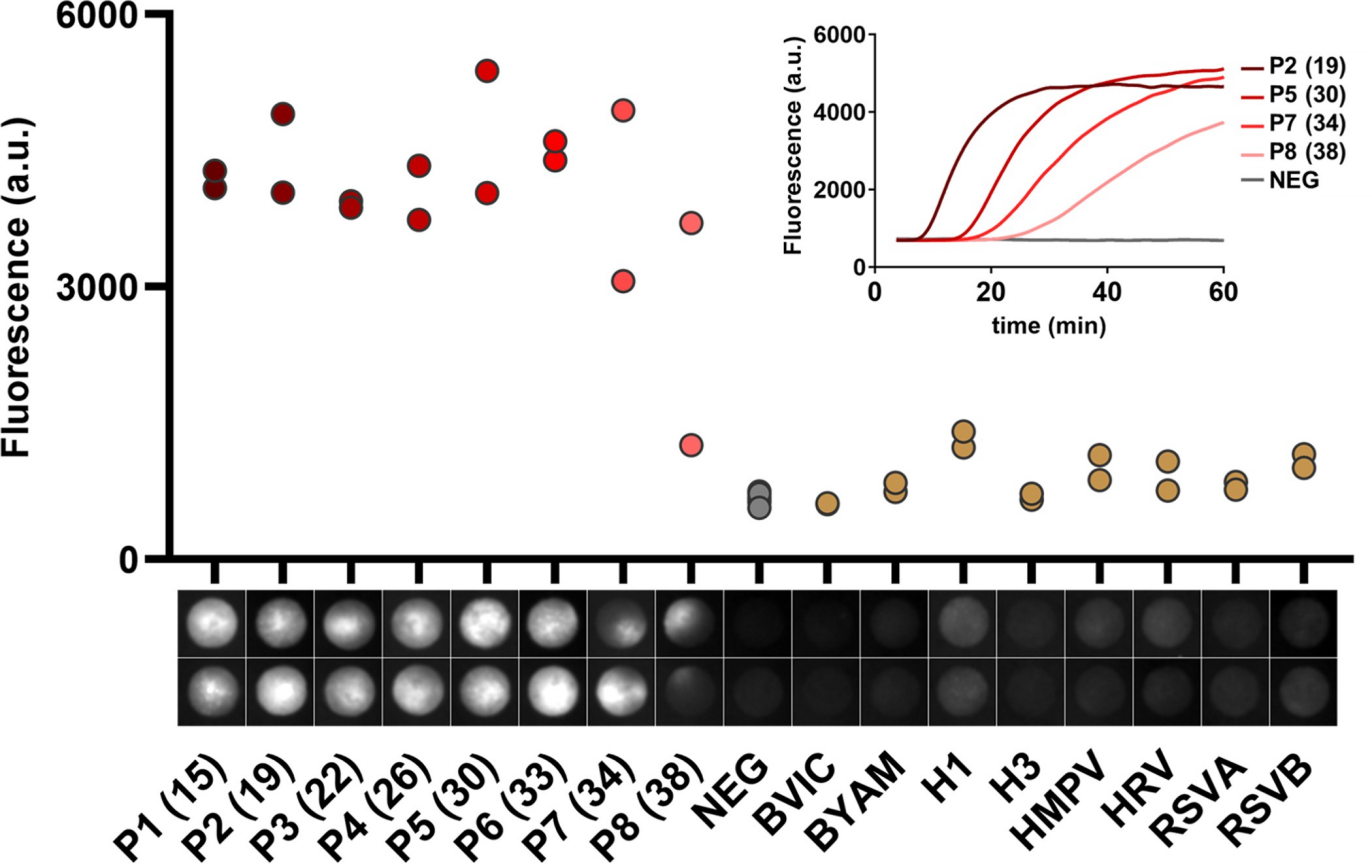
Detection of SARS-CoV2 in clinical samples, analytical performances of the test: Analytical sensitivity and Specificity end point (t = 60 min) measurements obtained for SARS-CoV-2 positive samples P1-P8 (SARS-CoV-2 RT-PCR Ct values in parenthesis), negative samples (individuals with a negative SARS-CoV-2 RT-PCR) and, on the right part, patients diagnosed with other respiratory infections. The low intensity level measured on the negative samples (grey dots) corresponds to background fluorescence. The internal controls (RNA 18s) are displayed in S1 Fig in S1 File. At the bottom, series of disk images obtained at the end point, each vertical pair corresponding to duplicate assays. (Insert) RT-LAMP amplification curves obtained by real-time monitoring of the fluorescence produced by an intercalating dye (SYTO-82).

Fig 2 shows end-point results, obtained with the device of Fig 1, sixty minutes after sample introduction, along with, in the insert, the kinetics of five samples. Below the abscissa of Fig 2, we show the fluorescence images of the reaction disks at t = 60 min. Heterogeneities of the fluorescence field are visible on most of them, a phenomenon we attribute, speculatively, to heterogeneous nucleation, cluster formation of amplicons and low diffusion of these clusters inside the porous medium. The fluorescence intensities level, plotted on Fig 2 are the averaged value of the 25% brighter pixels within the reaction disk. We consider that a sample is positive if the fluorescence signal is at least twice the average noise level, which is approximately 800 arbitrary units in our system.

As shown by Fig 2, Patient samples 1–7, i.e up to a a viral load of 31 copies per microliter of sample, which corresponds to a RT-PCR Ct value of 34. The sample n°8, detected at a Ct value of 38 by RT-PCR (1 copy per microliter of sample), is also detected in the upper disk, but ambigously in the lower one. We conclude that the limit of detection of the method is around 1 copy per microliter of initial sample (i.e. detected at a Ct value of 38 by RT-PCR). Such an analytical sensitivity is comparable to real-time RT-PCR performances. These conclusions do not sensibly depend on the image treatment. For instance, the value of 25% we took above, for determining the intensity levels on Fig 2, is not critical. We varied this number between 10 and 50%, without significant change in the results. Sup Mat 4 shows a classification based on the fluorescence standard deviation, which leads to the same results. Naked eye could also be used to distinguish positive from negative samples. The method of treatment of the images is thus not critical.

The insert of Fig 2 shows the kinetics of five RT-LAMP reactions, for different viral load levels, using SYTO82 as fluorescent intercalating agent. The RT-LAMP reaction kinetics obtained on a sample from a healthy individual has been represented, for estimating the background noise [31]. This noise level corresponds to the natural fluorescence of the reaction disk, i.e approximately 800 arbitrary intensity units. The fluorescence intensities of the SARS-CoV-2-positive samples are located well above. They have the expected sigmoid shape. The sample with the highest viral load, (presenting a Ct value of19, equivalent to 8x10^6^ genome copies per microliter of sample), was detected after 15 minutes. At higher Ct values, i.e. as viral load decreases, the signal takes off at later times. Plateaus are observed for the patient sample pools 1–6, i.e up to a Ct value of 33. For the samples with the lowest viral loads (pools 7 and 8), i.e., 31 and 1 genome copies per microliter, a plateau is not fully reached, but still, the virus can be detected without ambiguity.

The right part of Fig 2 addresses the question of the specificity. The images show that for non-SARS-CoV-2 pathogens, in almost all cases, fluorescence is barely visible. Sample H1 is seen above the background, for unclear reasons. However, quantitatively, the difference with the average background is 20%, which is not significant. Fig 2 shows that the specificity of the test, based on this set of pathogens, is 100%.

**Fig 3:**
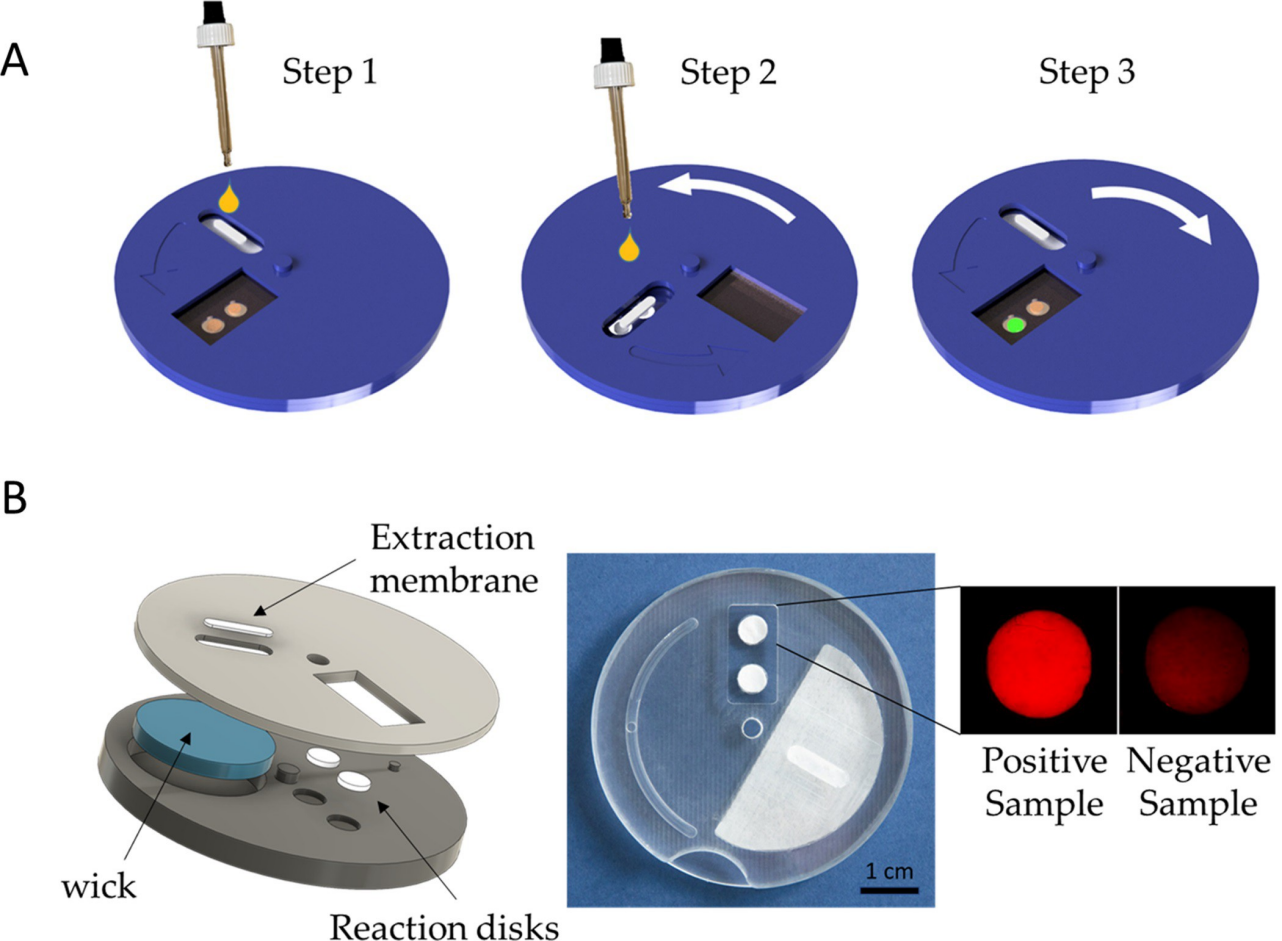
Description of the COVIDISC. A–COVIDISC workflow decomposed in three steps (the three steps include the six steps of Fig 1, in the same the workflow): 1 –injection, washing (fluids flow through the capture membrane and get absorbed by capillarity in the absorbent wick (in blue)), drying. 2—Disk rotation and elution; 3 –Disk counter-rotation, coverage of the reaction zone by a PCR sealing film, heating, amplification and readout. B–Left: Exploded structure of the device. Center: Picture of a prototype. Right: QUASR readout photograph of a test on RNA extracts of SARS-CoV-2, processed as in Fig 3A; (left) Positive sample; (right) negative sample.

In order to transform the foldable paper system into a practical POC device, we created the “COVIDISC”, shown in Fig 3.

Fig 3A shows the COVIDISC workflow, which exactly reproduces that of Fig 1B (see caption of Fig 3A). The structure of the device, shown in Fig 3B consists of two plastic disks, 5 cm in diameter, able to rotate around a common axis. The extraction membrane, the wick and the reaction disks are force fitted. By performing rotations, injections and heating, one executes the workflow of Fig 1B (see S2 Table in S1 File). On adding QUASR probes (Quenching of Unincorporated Amplification Signal Reporters) [32] to RT-LAMP, we obtain a naked eye YES/NO answer. Fig 3B (right) shows the readout of RNA extracts of SARS-CoV-2, captured with the camera of a smart phone. We used an oven to maintain the temperature at 65°C and one LED and two gelatin filters to take the picture (see S4 Fig in S1 File). The equipment needed for running the test is thus minimal.

## Conclusion

To the best of our knowledge, the system we present here is unique. No such assay, i.e. portable, low-cost, including NA extraction/elution, user friendly and with performances comparable to RT-PCR, has been reported in the literature thus far. The results presented here have been obtained with 21 clinical samples and larger sets of samples are certainly desirable. Nonetheless, our results indicate that, with a minimal equipment, one can extract, wash, elute, reverse-transcribe, amplify and measure the kinetics, with a LoD comparable to the gold standard real-time RT-PCR, i.e. 1 genome copies per microliter of clinical sample, and a specificity, of 100%, based on the set of pathogens we have used. We also created a portable device (COVIDISC) that can be used at the point of care. The production cost we estimated for such a device is around 2–4 $. This type of device is thus appropriate for ressource-limited countries but also for developed countries, to reduce the now considerable costs generated by the generalization of RT-PCR testing in the population. Generalizing these tests at the doctor’s practice, at the working place or in pharmacies could allow the isolation of infected patients without delay, shortening their quarantine, reducing logistics and costs and offering a new efficient approach to manage the pandemics. In the future, obviously, the same technology could be used for other pathogens.

## Supporting information

S1 File The supplementary information file contains information about the primer sequences (S1), the internal positive controls (S2), the protocol of operation for the COVIDISC (S3), the signal processing used (S4), the relation between the Ct and the viral load (S5) and the COVIDISC design (S6). (DOCX)
