# Supplementary material for: Performing point-of-care molecular testing for SARS-CoV-2 with RNA extraction and isothermal amplification
Source: PLoS One. 2021 Jan 11;16(1):e0243712. doi: 10.1371/journal.pone.0243712 (PMC7799764; doi:10.1371/journal.pone.0243712)
Supplement: S1 File — (DOCX) [file pone.0243712.s001.docx]

1. **RT-LAMP primers sequences**

| Primer | Sequence (5’ to 3’) |
| --- | --- |
| Covid-19_F3 | TCCAGATGAGGATGAAGAAGA |
| Covid-19_B3 | AGTCTGAACAACTGGTGTAAG |
| Covid-19_FIP | AGAGCAGCAGAAGTGGCACAGGTGATTGTGAAGAAGAAGAG |
| Covid-19_BIP | TCAACCTGAAGAAGAGCAAGAACTGATTGTCCTCACTGCC |
| Covid-19_LoopF | CTCATATTGAGTTGATGGCTCA |
| Covid-19_LoopB | ACAAACTGTTGGTCAACAAGAC |
| 18S_rRNA_L3 | GTTCAAAGCAGGCCCGAG |
| 18S_rRNA_B3 | CCTCCGACTTTCGTTCTTGA |
| 18S_rRNA_FIP | TGGCCTCAGTTCCGAAAACCAACCTGGATACCGCAGCTAGG |
| 18S_rRNA_BIP | GGCATTCGTATTGCGCCGCTGGCAAATGCTTTCGCTCTG |
| 18S_rRNA_LoopF | AGAACCGCGGTCCTATTCCATTATT |
| 18S_rRNA_LoopB | ATTCCTTGGACCGGCGCAAG |

**Supplementary table 1 :** Sequences of the primers used in the SARS-CoV-2 test (From Lamb et al (19,28))

1. **Internal positive controls analysis**

In our device, each clinical sample is split into two reaction disks: a SARS-CoV-2 test and the human 18S ribosomal RNA test. This endogenous gene serves as internal positive control to monitor: (i) sample quantity and quality; (ii) RNA extraction efficiency; (iii) freeze-dried reagents stability; (iv) RT-LAMP amplification efficiency (associated with the proper functioning of the heater). The result of the SARS-CoV-2 test should be rejected if the internal control test is not positive.

For each sample (data point) presented in Figure 2, we ran the internal control on the same device, with a RT-LAMP mix detecting human 18S ribosomal RNA. The histogram of these 39 data points is represented on Figure S2B. The minimal signal of all these controls (2312 a.u.) is well above the average value of the no template controls (703 a.u. ± 52 a.u, 5 replicates). (standard deviation)) (See Fig S3) . The histogram is substantially broad; it may reflect the fact that the amount of human material, and thereby that of 18S RNA quantity, may vary from one sample to the other.

**
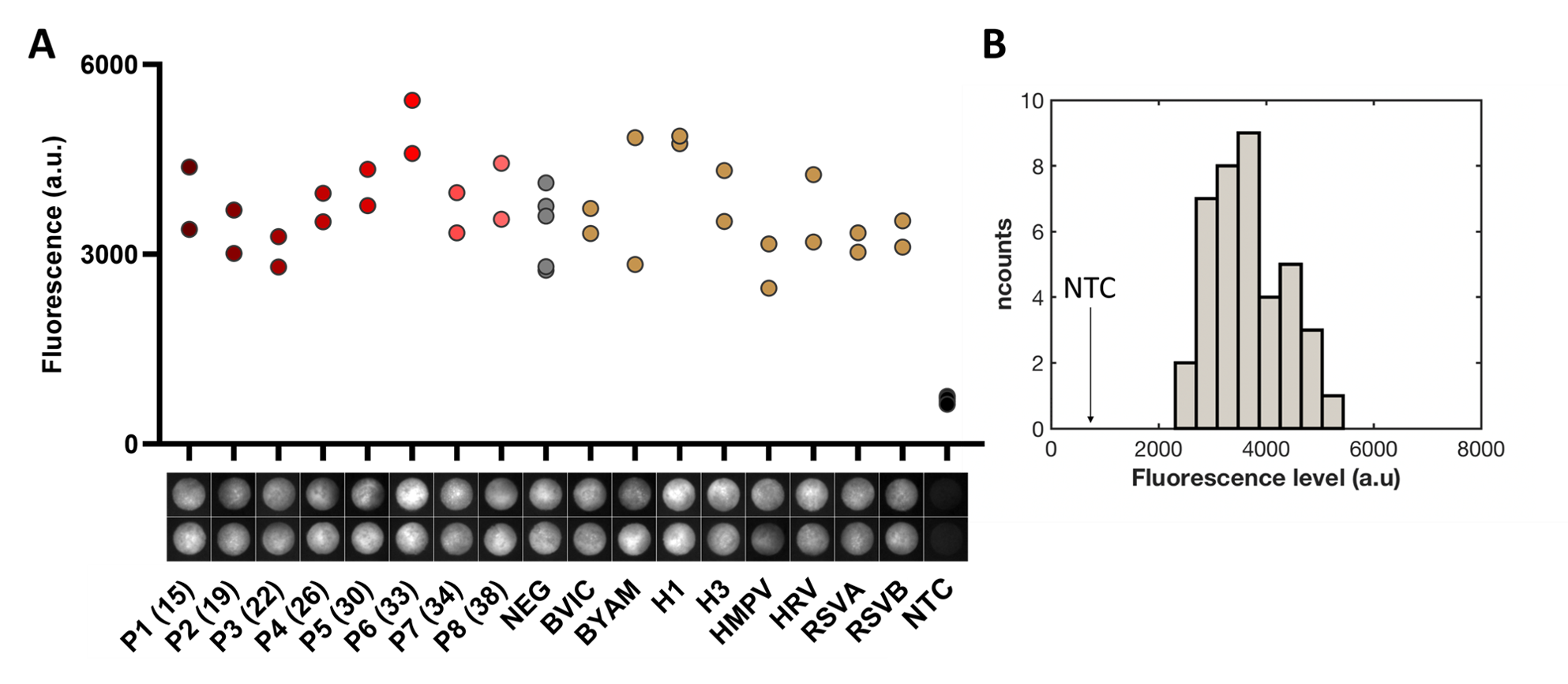
**

**Fig. S1:** A – Human 18S RNA internal control on naso-pharyngal samples: end point (t=60 min) measurements of each internal control disk respectively corresponding to Fig.2 tests disks. Black dots display no template controls (NTC), 5 replicates. B – Frequency histogram of the fluorescence gray levels of the 39 human 18S RNA internal controls, with a class interval of 390 units. Each level is the averaged intensity of the 25% brighter pixels of the end point image. The average fluorescence level of the no template controls (NTC) located at a level of 703 is shown on the graph.

1. **List of operations to be realized to perform the test on the COVIDISC**

| N° | Description |
| --- | --- |
| 1 | Inject the lysed sample onto the extraction membrane |
| 2 | Inject the washing buffer to wash the membrane |
| 3 | Wait a few minutes for drying |
| 4 | Turn the upper disk by 90°to place the extraction membrane onto the reaction disk |
| 5 | Inject the elution buffer to elute onto the reaction risk |
| 6 | Turn the upper disk by 90° in the opposite direction |
| 7 | Cover the reaction disk with a sealing PCR film (an operation that can be suppressed by modifying the design) |
| 8 | Place the COVIDISC in an oven at 65°C for ~ 45 min -1h |
| 9 | After returning to room temperature, read the fluorescence with the naked eye or a cell phone using appropriate filters |

**Supplementary Table 2 :** Covidisc operating mode

1. **Experimental set-up to record fluorescence and signal processing**

We used a dedicated set-up to acquire fluorescence, based on a Leica Z16 APO macroscope, equipped with FAM and Texas red cubes, and recorded images using an EM-CCD camera from Hamamatsu. This set up acquired an image of the reaction disks placed on a heating plate (65°C) every 10 seconds.

Reaction disks were detected using active contours to automatically delineate their shape, using an image of the device taken before heating/starting the amplification. A single seed point inside each disk needs to be provided to perform each contour estimation. The Icy image analysis platform ([www.icy.fr](http://www.icy.fr/)) was used to extract the data. The fluorescence signal plotted on Figure 2 is the average value of the 25% brighter pixels within the reaction disk, a simple approach to take into account profiles of low viral load samples (presenting high RT-PCR Ct values), where the reaction happens and results in very bright pixels only in a subpart of the disk. We explored whether the heterogeneity of the fluorescence within the reaction disks could be used to reinforce the classification. We found out that the standard deviation (SD) of the fluorescence intensity within the reaction disk could increase the contrast between COVID-19 pools and negatives/other viruses samples (Figure S4). A SD threshold can thus classify all COVID-19 pools as positive and the negatives/non-specific samples as negative. We will explore in further experiments whether this parameter could complete/replace the fluorescence intensity to better classify tests as positive or negative.


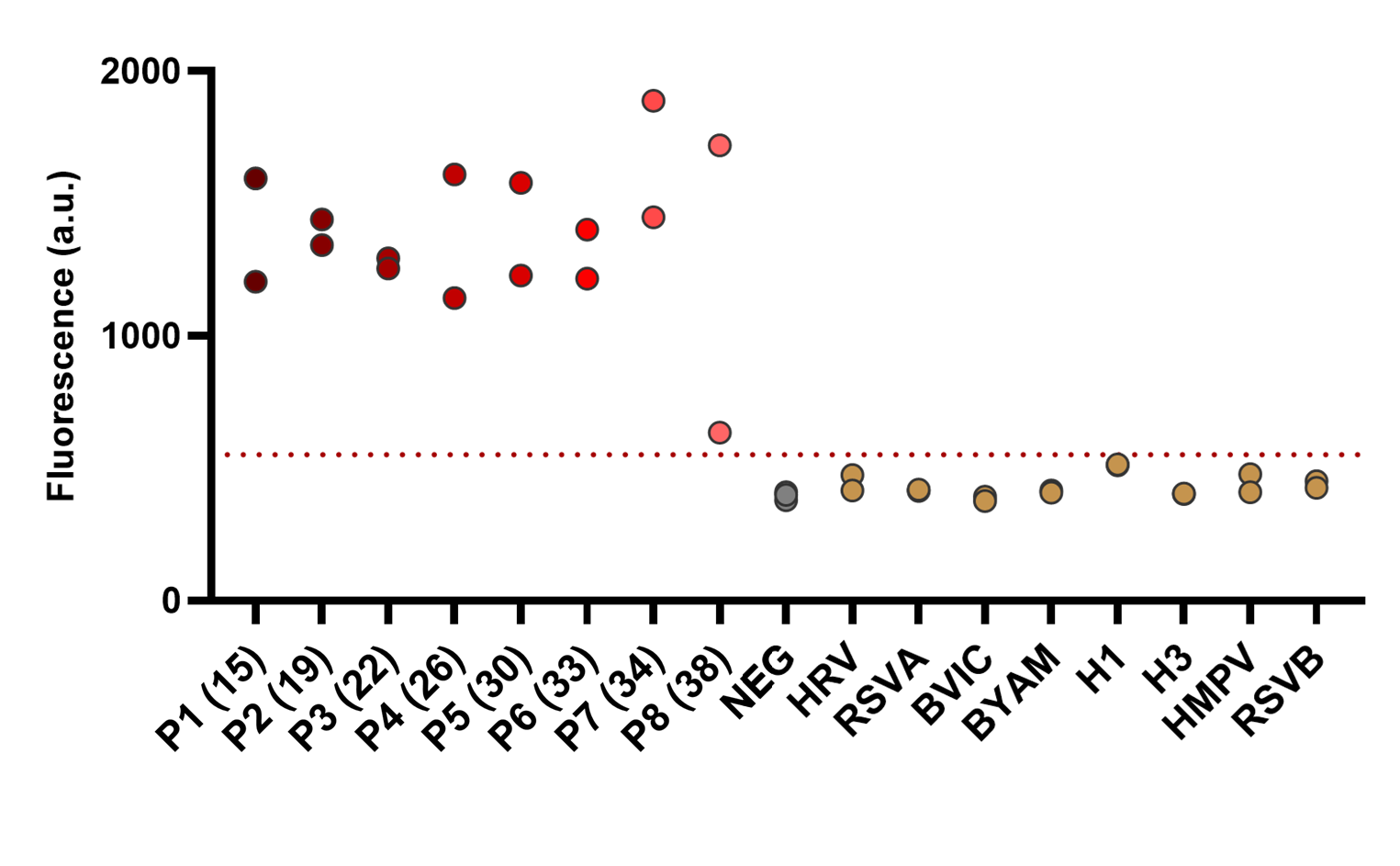


**Figure S2** : Sensitivity and Specificity measurements for SARS-CoV detection. Same experiment and data as in Figure 2, where the signal is the standard deviation within the disk on the last (t=60min) picture. Red dotted line separating all positive from negative samples stands at SD = 550.

1. **Calibration Ct – Viral load, based on IP2 qRT-PCR**


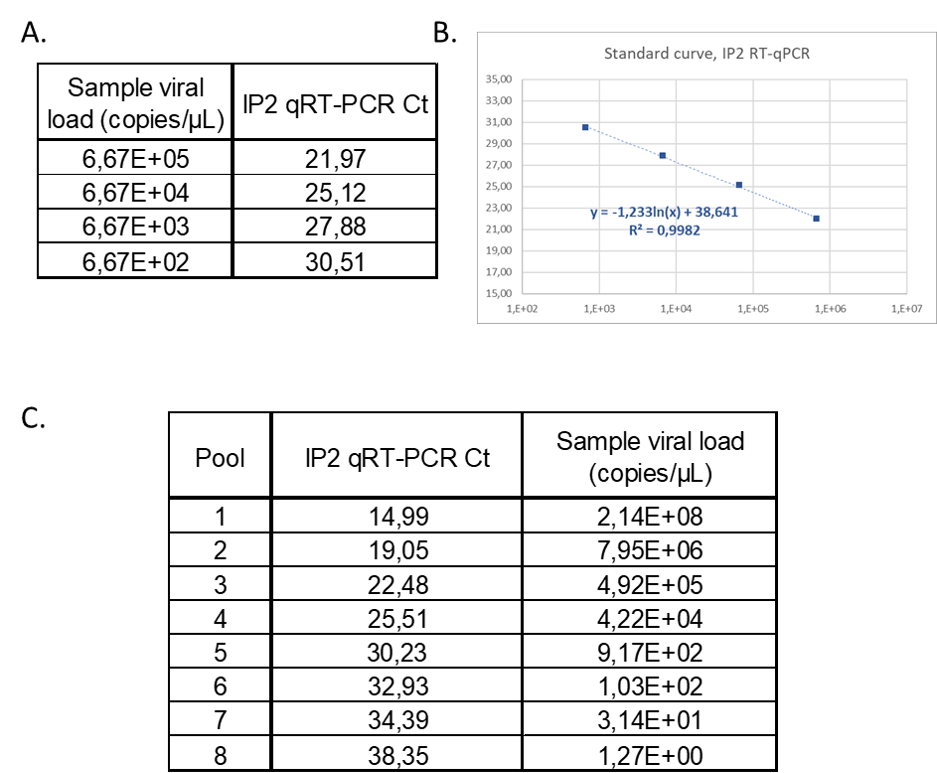


**Figure S3** : Extrapolation of CNR pools viral load. (a) Ct associated to known standard dilutions of SARS-CoV-2 transcripts, taking into account the dilution steps for PCR testing and sample extraction (factor 15 in total). (b) Associated Standard curve and logarythmic fit. (c) SARS-CoV-2 viral load for each of the 8 pools. Number of copies per microliter of sample is deduced from the logarythmic fit.

1. **On-field acquisition set-up.**


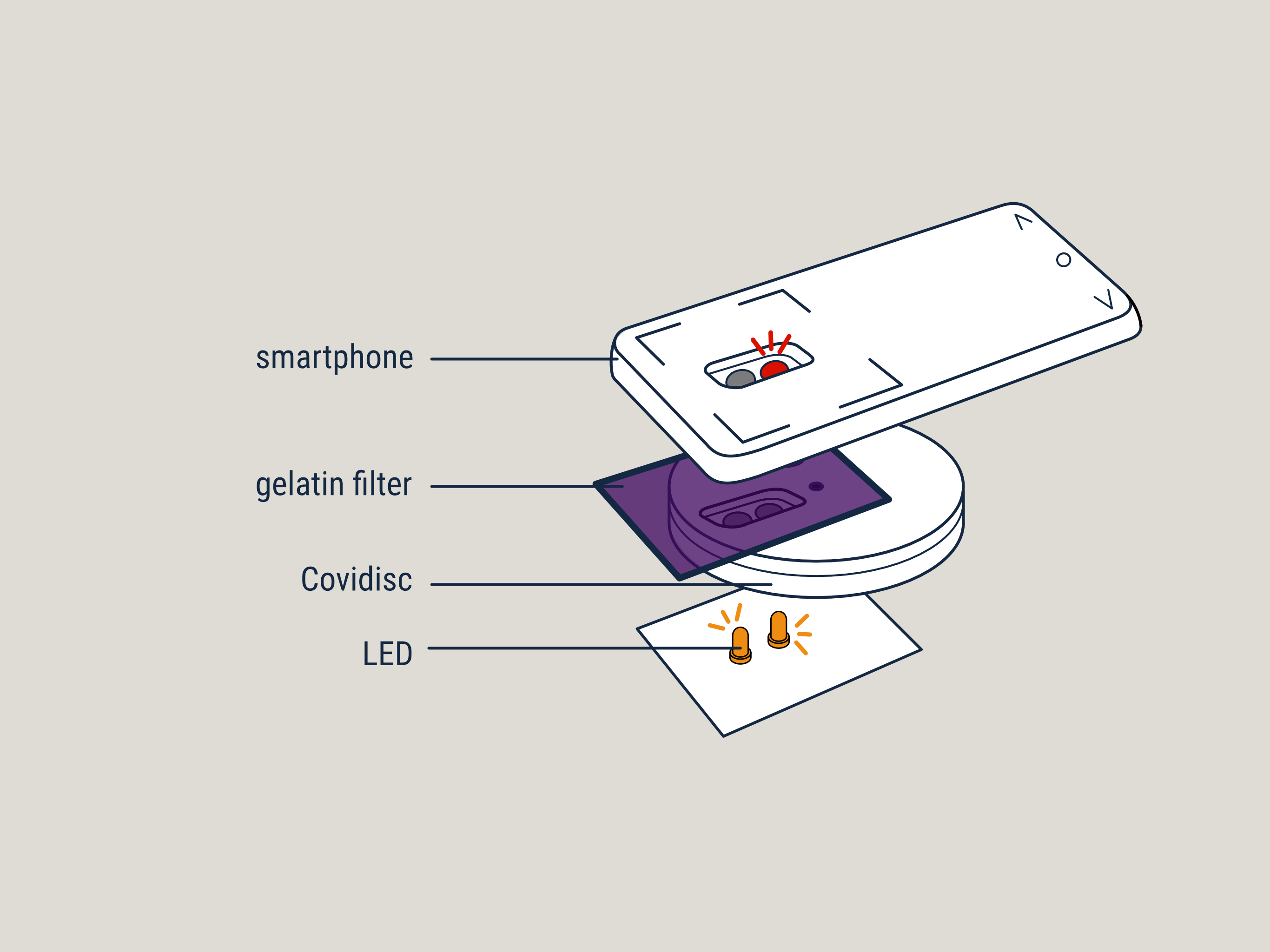


**Figure S4-1** : Sketch of the setup used in Fig 3 for the naked eye or cell phone observation.

To propose an end-point measurement, utilizable in the field, with Texas Red fluorescent probes, we built a set-up, composed of 2 LEDs (main wavelength at 560nm, RadioSpare 809-1754) disposed at the bottom of the COVIDISC (see Fig S4-1). A photographic gelatin filter (Lee filter, color filter 797) was used to filter excitation wavelength and get emission signal transmitted by the reaction membrane. We can then image the fluorescence with a smartphone camera (we used here a Samsung galaxy 8 device, ISO 400, focal ratio 1/90)

A version of a practical device is represented in Fig S4-2, the illumination system being embodied in a plastic box. On the Figure, we can see the COVIDISC, the heating device (in form of a toaster) and the illumination device, based on the scheme of Fig S4-1. This version provides a concrete view of the system, but it will certainly evolve, or generate different sub-versions, depending on the needs expressed by users.


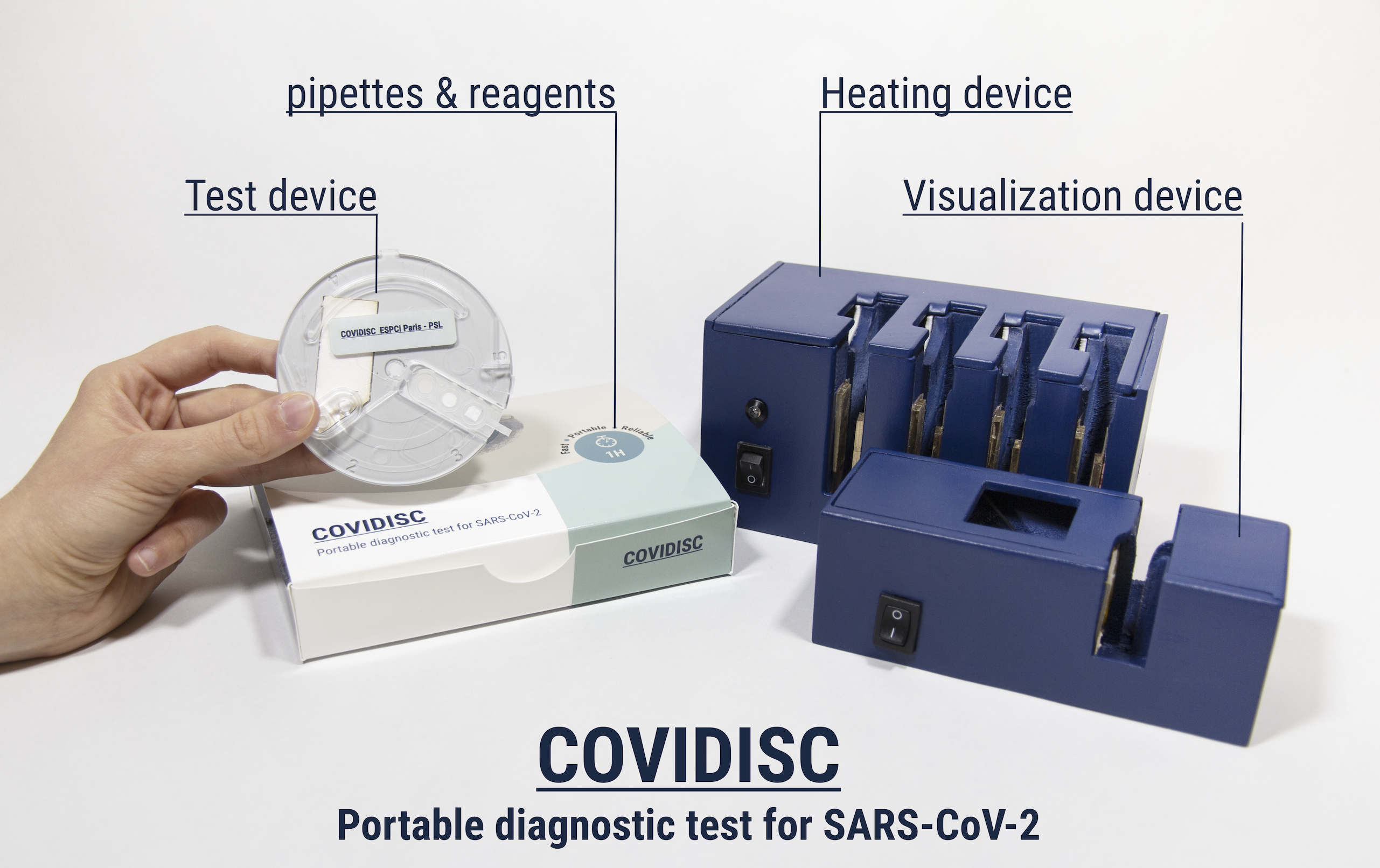


**Figure S4-2** : A realization of the COVIDISC system, including the test, the heater and the visualization setup.
